# Nanoporous mannitol carrier prepared by non-organic solvent spray drying technique to enhance the aerosolization performance for dry powder inhalation

**DOI:** 10.1038/srep46517

**Published:** 2017-05-02

**Authors:** Tingting Peng, Xuejuan Zhang, Ying Huang, Ziyu Zhao, Qiuying Liao, Jing Xu, Zhengwei Huang, Jiwen Zhang, Chuan-yu Wu, Xin Pan, Chuanbin Wu

**Affiliations:** 1 School of Pharmaceutical Sciences, Sun Yat-Sen University, Guangzhou 510006, China; 2 Shanghai Institute of Materia Medica, Chinese Academy of Sciences, Shanghai 201203, China; 3 Department of Chemical and Process Engineering, University of Surrey, Guildford, Surrey GU2 7XH, United Kingdom; 4 Zhongshan WanYuan New Drug R&D Co., Ltd., Zhongshan City 528451, China; 5 Guangdong Research Center for Drug Delivery Systems, Guangzhou 510006, China

## Abstract

An optimum carrier rugosity is essential to achieve a satisfying drug deposition efficiency for the carrier based dry powder inhalation (DPI). Therefore, a non-organic spray drying technique was firstly used to prepare nanoporous mannitol with small asperities to enhance the DPI aerosolization performance. Ammonium carbonate was used as a pore-forming agent since it decomposed with volatile during preparation. It was found that only the porous structure, and hence the specific surface area and carrier density were changed at different ammonium carbonate concentration. Furthermore, the carrier density was used as an indication of porosity to correlate with drug aerosolization. A good correlation between the carrier density and fine particle fraction (FPF) (*r*^*2*^ = 0.9579) was established, suggesting that the deposition efficiency increased with the decreased carrier density. Nanoporous mannitol with a mean pore size of about 6 nm exhibited 0.24-fold carrier density while 2.16-fold FPF value of the non-porous mannitol. The enhanced deposition efficiency was further confirmed from the pharmacokinetic studies since the nanoporous mannitol exhibited a significantly higher AUC_0-8h_ value than the non-porous mannitol and commercial product Pulmicort. Therefore, surface modification by preparing nanoporous carrier through non-organic spray drying showed to be a facile approach to enhance the DPI aerosolization performance.

The local delivery of drug to the lung can be achieved by using nebulizers, pressurized meter dose inhalers (pMDIs), or dry powder inhalers (DPIs). Among the three inhalations, DPIs offer advantages over nebulizers and pMDIs from the environmental and economical points, since they are propellant-free, portable, and stable as a result of dry state^1^. These advantages make DPIs particularly suited to therapeutic aerosols.

The success of therapy using DPIs depends on their ability to delivery active pharmaceutical ingredients (APIs) to the deep lung. In order to improve the metering, handling, and ability to deliver APIs, the APIs are frequently formulated with major coarse carrier particles as adhesive mixtures^2^. The adhesion of particles is a surface phenomenon and therefore, the drug-carrier adhesion properties are deeply affected by the surface morphology of carrier particles^3^. Modifying particle surface roughness has a significant impact on the interparticulate forces of the adhesive mixtures and further the drug aerosolization performance^4^,^5^. The improvement of drug aerosolization was dependent on the scale of surface roughness by altering the drug-carrier contact area. A consensus has been reached that the carriers with small asperities showed the potential to enhance drug aerosolization by reducing the interparticulate forces and increasing drug detachment from the carriers.

As the surface roughness largely affects particle-particle interactions and hence aerosolization performance, different attempts to modify the surface roughness of carrier particles to achieve lower adhesion forces and consequently improved inhalation performance were reported. These attempts mainly include particle crystallization with different solvents^6^, adding fine particles to alter the surface roughness by occupying the active sites^7^, particle milling with different time and material^8^, polymer coating of particles^9^, and spray drying at different drying conditions^10^. However, the crystallization method has the demerits of using organic solvents, multiple steps and unpredictable physicochemical properties. Utilization of fine particles is fraught with the risk of falling off the coarse carrier particles and consequently reducing the effectiveness. For the milling and coating method, it is difficult to control the microstructure of particles, and even it is easy to cause inter- and intra- batch variability^4^.

The common characteristic lying behind the documented methods was to design carriers with small surface asperities. Carrier with such a microstructure meets the requirements for powder particles used in inhalation systems, since it can reduce drug-carrier adhesion forces and ease drug re-entrainment from carriers to allow for tiny airway deposition. The small surface asperities were composed of small crevices, hollows or other trapps. From this point of view, the nano-metered pores were supposed to create small surface asperities for carriers. The pore in a size range below 200 nm was defined as nano-metered pore^11^. Up till now, there has been no report on surface roughness modification of carrier by preparing porous carrier with nano-metered pores (nanoporous carrier). Moreover, the pores in nanoscale size could confer major advantages to the carriers, including reduced density and decreased impact strength, which were favorable for improving DPI aerosolization performance^11^,^12^,^13^,^14^,^15^. Therefore, nanoporous carrier was exploited to enhance drug deposition efficiency and attain better aerosolization performance by constructing small surface asperities through the nano-metered pores.

The methods to prepare nonoporous particles have been well documented in previous researches^11^,^12^,^13^,^16^,^17^. However, the materials used for producing nonoporous particles were commonly inorganic or nonbiodegradable materials, which were not allowed for application as inhalable particles. There are currently few studies providing the method to prepare biodegradable hydrophilic nanoporous particles. Among these methods, spray drying is the most potential technique for scale and continuous production. The common problem arose from current spray drying method was using organic solvent and/or additives, which potentially cause safety hazards. For example, Iskandar *et al*. used a two-nozzle spray drying system to produce porous hyaluronic acid particles^18^. The suspension containing precursor and templating agent (polystyrene latex) was initially spray-dried to produce composite particles, followed by washing with large quantities of organic solvent to remove the templating agent. Ógáin *et al*. produced nanoporous microparticles of trehalose and raffinose by spray drying from a solvent-antisolvent system^19^, such as methanol- n-butyl acetate. The differences in solvent volatization can enhance the pore formation of the particles. To date, there has been no report providing a non-organic solvent spray drying technique to prepare porous particles. The avoidance of using organic solvents precludes the potential safety hazards.

Therefore, the objective of this study was to initiate a non-organic solvent spray drying technique to prepare nanoporous mannitol with small asperities as DPI carrier for enhanced aerosolization performance. The formation mechanism of nanoporous mannitol was exploited by investigating the effect of key factors, including the outlet temperature and concentration of ammonium carbonate, on the pore formation of mannitol. Furthermore, the spray-died mannitols were used as model carriers to establish the correlation between carrier density and aerosolization performance since the carrier density except other characteristics was changed by altering the concentration of ammonium carbonate. Finally, the pharmacokinetic profiles on the DPI formulations with nanoporous and non-porous mannitol as carriers, as well as Pulmicort were compared to confirm the feasibility of using nanoporous carriers to enhance *in vivo* aerosolization performance of DPI.

## Results and Discussion

### Characterizations of spray-dried mannitols

#### Morphology

**Figure 1:**
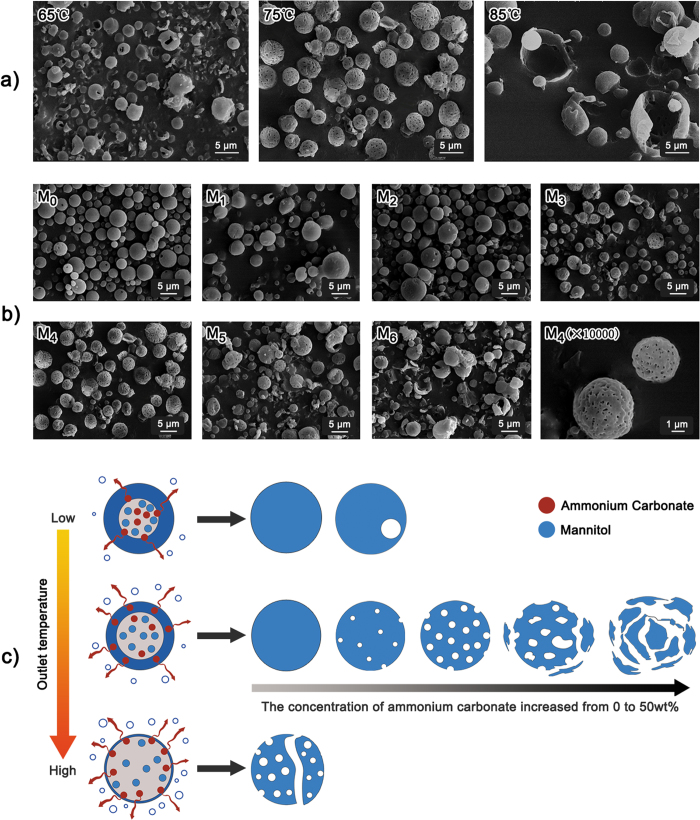
The SEM micrographs and formation mechanism of producing nanoporous mannitol as a function of outlet temperature and ammonium carbonate concentration: (**a**) the SEM micrographs of mannitols produced at different outlet temperature, (**b**) the SEM micrographs of mannitols as a result of ammonium carbonate concentration, and (**c**) the formation mechanism of nanoporous mannitol. M_0_–M_6_ were referred to the spray-dried mannitols prepared at the ratio of ammonium carbonate: mannitol = 0, 1:7, 1:5, 1:4, 1:3, 1:2, and 1:1, respectively. The corresponding ammonium carbonate concentration (wt%) was 0, 12.5, 16.7, 20, 25, 33.3, and 50.

The morphology of spray-dried mannitols produced from non-organic solvent system was shown in Figure 1. For this non-organic solvent spray drying technique, the outlet temperature and the concentration of ammonium carbonate were shown to be key factors influencing the pore formation of mannitols. Regularly porous mannitols were successfully prepared at the outlet temperature of 75 °C and 25 wt% ammonium carbonate. Therefore, the non-organic solvent spray drying technique provided a feasible access to prepare porous particles since the key parameters were easily controllable.

Figure 1a showed the morphology of mannitols produced at different outlet temperatures. Regularly porous mannitol was only obtained at 75 °C, shattered and fractured mannitol was observered at 85 °C, and spherical but almost no porous mannitol was found at 65 °C. Figure 1b displayed the morphology of mannitols produced at different ammonium carbonate concentrations. M_0_–M_3_ exhibited spherical but non-porous morphology, while M_4_–M_6_ showed porous morphology. It was suggested that porous mannitols could be obtained only when a certain ammonium carbonate concentration (≥25 wt%) was exceeded. Interestingly, all mannitols except M_0_ had a rough surface, indicating that the surface roughness of particles could be increased by adding ammonium carbonate during spray drying.

Based on these facts, the mechanism of producing porous mannitol is illustrated in Figure 1c. As soon as the droplet enters the heat flow, water evaporation takes place at the surface of the droplet. Then the concentration of solute at the surface increases with solvent/volatile evaporation, causing the diffusion of solute towards the center of droplet. Therefore, the particle formation may be a balance between solvent/volatile evaporation and solute diffusion^20^. This procedure would be discussed below from the aspects of outlet temperature and ammonium carbonate concentration

Initially, the influence of temperature was taken into account. With higher temperatures, the increase in surface concentration was much faster and shell formation takes place at an earlier instant, resulting in a lower solute concentration at the surface. In this case, the shell wall is thinner and more prone to be broken through by the volatization. Additionally, the evaporation rate was higher, and a pressure might form inside the particle, once larger enough, which will lead to the rupture and subsequent collapse of the particle shell, resulting in irregular shape of particles^10^. Therefore, porous shattering and fracture of mannitol were evident at 85 °C. At lower temperatures, the diffusion is significantly slower than the evaporation, which means a higher solute concentration and a thicker shell wall should be formed at the droplet surface. Accordingly, almost no porous mannitol was found at 65 °C. The porous mannitol obtained at 75 °C might be a result of a good balance between solvent evaporation/volatile and solute diffusion.

Furthermore, the influence of ammonium carbonate concentration on producing porous mannitol was investigated since it affected the solvent/volatile evaporation. It was postulated that the thickness of shell wall was constant at a specific temperature. More volatile was generated with the increase of ammonium carbonate concentration, and would cause the inflation of droplet and facilitate solvent evaporation. Non-porous mannitol could be formed when the force induced by solvent/volatile evaporation was insufficient to break through the shell wall. However, one or several occasional pores could be generated in the mannitol (M_0_) when some weak films occurred in the wall. On the contrary, porous mannitol could be formed with the increased ammonium carbonate concentration, which generated sufficient force of solvent/volatile evaporation to pierce through the shell wall. However, it is worth mentioning that the larger force driven by faster solvent/volatile evaporation would accelerate the collapsion of wall, and lead to the fusion of pores. Finally, fused or collapsed particles, as well as less pores were found in M_5_ and M_6_ as compared to M_4_.

Actually, the geometries of the spray dryer were also critical factors influencing the morphology of mannitols^21^. However, the spray dryer in a lab scale employed in this study was fixed and further studies would be focused on the impact of those geometries.

#### Particle size, bulk and tap density

Table 1 displayed the particle size, bulk and tap density of the spray-dried mannitols. As compared to the mannitol spray-dried alone (M_0_), the particle size of co-spray dried mannitol (M_1_–M_6_) remarkably increased, while slightly increased with the increasing amount of ammonium carbonate. The increased particle size for M_1_–M_6_ may be attributed to the inflation of droplets resulted from ammonium carbonate decomposition with volatile during spray drying. However, the extent of droplet inflation was probably limited, so the particle size of M_1_–M_6_ was approachable. Besides, mannitols (M_1_–M_6_) demonstrated considerably lower bulk density than M_0_, and M_4_ exhibited the lowest bulk and tapped density. Previous researches have demonstrated that particle density was related to the internal structure^22^ and geometric size^2^,^23^ of particles. Thereby, this result was partly ascribed to the porous/hollow structure of M_1_–M_6_, which could reduce interparticulate forces and aggregation between particles to make particles less condensed, leading to a lower bulk density. Further, M_1_–M_6_ had a larger geometric size and hence a fewer number of particle-particle contact points with neighboring particles so that they became loosely packed and had a considerably lower particle density.

#### Specific surface area and pore size

As shown in Table 1, an increase in specfic surface area (SSA) of M_1_–M_6_ was observed as compared to M_0_, and the magnitude was dependent on the concentration of ammonium carbonate. The SSA initially increased with the increasing concentration of ammonium carbonate, then dropped when the concentration of ammonium carbonate reached to 25 wt% of total solid content. The change tendency in the SSA of mannitols was consistent with the porosity and pore size of mannitols, as shown in SEM micrographs (Figure 1), which was well agreed with previous documents^24^,^25^. The pore size resulting from different ammonium carbonate concentration was discussed in the part of aforementioned formation mechanism of porous particles. According to Zellnitz S *et al*., SSA is also an indication of surface roughness, the larger SSA indicative of the larger surface rougness^26^. The degree of surface roughness could also be observed from the SEM photographs. Mannitols (M_1_–M_6_), especially M_4_ showed an obviously rough surface, while M_0_ had a smooth surface. Accordingly, a good correlation of SSA and surface roughness was achieved. Among all the mannitols, M_4_ showed a maximum SSA and a mean pore size of about 6 nm, thus was termed as nanoporous mannitol. The nano-metered pores was equated to the crevices, hollows or other trapps, making the porous mannitol form a small surface asperity to enhance drug deposition by reducing drug-carrier contact area and increasing drug-carrier detachment^27^.

#### Ammonium carbonate residues assay

**Figure 2:**
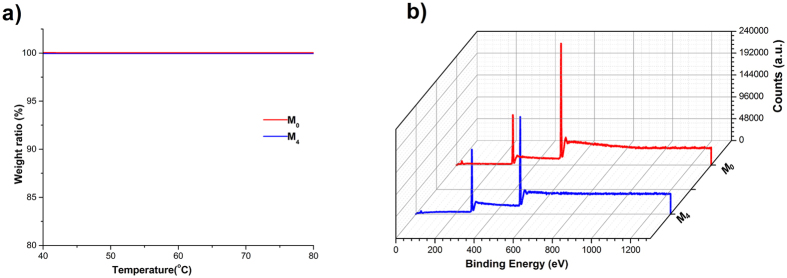
Determination of ammonium carbonate residue in the nanoporous mannitol (M_4_) and non-porous mannitol (M_0_): (**a**) TGA traces, and (**b**) XPS spectra.

Figure 2a showed that no change in the TGA traces of all mannitols between 40 °C and 80 °C was found, which suggested that no weight loss took place during heating to 80 °C. The TGA result indicated that no residual ammonium carbonate was present in the mannitols since the degradation of ammonium carbonate at the temperature of 62–70 °C would lead to weight loss.

The XPS measurement (Figure 2b) of elements in M_4_ and M_0_ showed that no N element was detected. This further confirmed the complete removal of ammonium carbonate during spray drying, since N element only existed in the ammonium carbonate. The absence of ammonium carbonate would reduce the risk of safety hazards induced by exogenous substance even though no information on the security issue of using ammonium carbonate was reported.

### *In vitro* and *in vivo* deposition studies

#### *In vitro* aerosolization performance

A key factor which affects DPI performance is the particle density of pure drug or drug loaded microsphere. However, there is still lacking information in the literature showing the effect of carrier density on the drug aerosolization performance^28^. Moreover, it is difficult to study one physicochemical property of the carrier on the drug aerosolization performance in isolation, since the modification of one variable generally results in a change in the other variables in the system^29^. Herein, spray-dried mannitols were firstly employed as model carriers to study the role of carrier density on the drug aerosolization performance since the other physicochemical properties of mannitols, including particle size, crystalline (data were shown in the supplementary information) and morphology were similar. Therefore, the effect of these properties on the aerosolization performance of the DPI formulations could be neglected, and the significant variation in carrier density (*P* < 0.05) was the major factor influencing the fine particle fraction (FPF).

**Figure 3:**
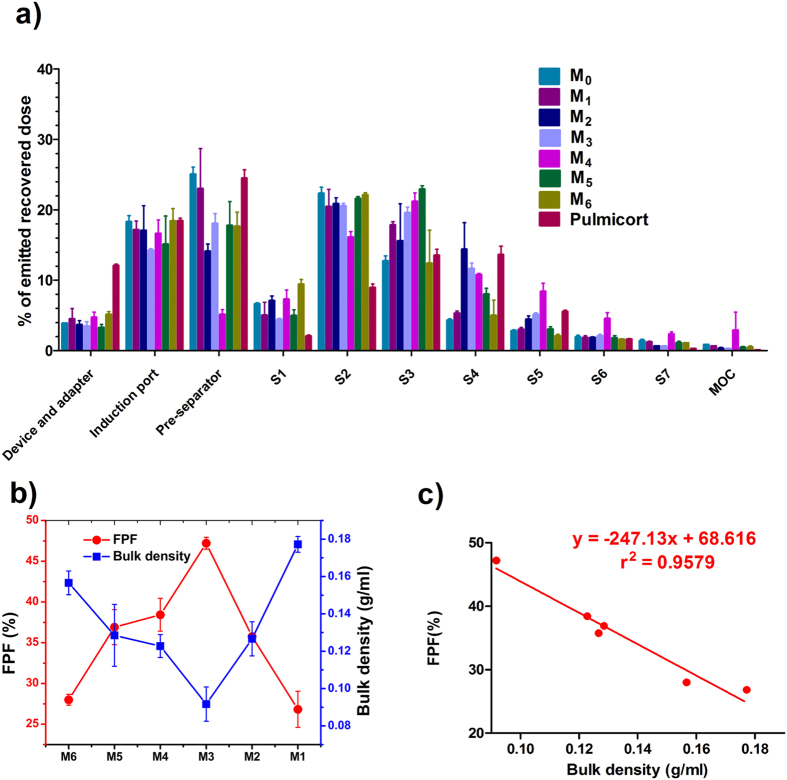
The influence of spray-dried mannitols with diverging particle density on the drug aerosolization performance withPulmicort as a comparision (*n* = 3): (**a**) NGI deposition profiles of budesonide after aerosolization by the Turbospin, (**b**) the bulk density of mannitols and FPF obtained from DPI formulations containing different mannitols as carriers, and (**c**) the linear relationship between bulk density of mannitols and FPF.

Budesonide mass deposition patterns of different DPI formulations were shown in Figure 3a. It could be seen that less drug deposited in the upper stages (before Pre-separator) for the carrier based DPI formulations than for the commercial product Pulmicort. However, the amount of drug deposited in the lower stages depended on the carriers. Consequently, the nanoporous mannitol (M_4_) exhibited the best aerosolization performance for DPIs and showed a 2.16-fold and 1.37-fold FPF value of non-porous mannitol (M_0_) and Pulmicort, respectively. This result could be achieved with the advantages of nanoporous particles. The nano-metered pores created small surface asperities for M_4_, which were intended to reduce the contact area and hence the interparticulate forces of drug and carrier particles^30^. As a result, drugs were more prone to detach from M_4_ and delivered to lower stages, resulting in a significantly high FPF value in the M_4_ formulation.

The variation in the bulk density and FPF for the spray-dried mannitols was displayed in Figure 3b. To correlate the drug aerosolization with carrier density, the inverse relationship (*r*^*2*^ = 0.9579) between bulk density and FPF was plotted in Figure 3c. It was demonstrated that a good correlation between bulk density and FPF was established, and the FPF of the DPI formulations increased with the decrease of carrier density.

### *In vivo* pharmacokinetic studies

**Figure 4:**
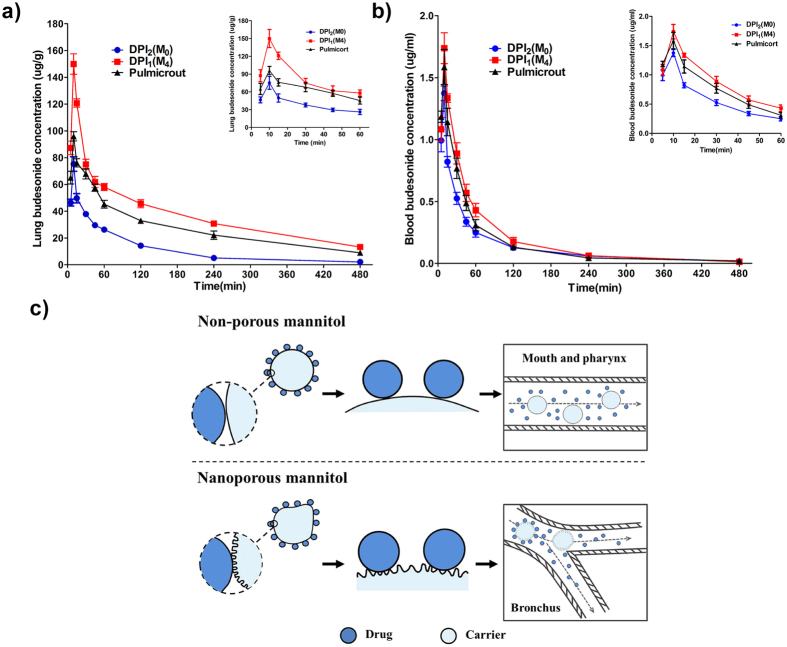
Budesonide concentration-time curve in the lung (**a**) and plasma (**b**) after administration of different DPI formulations (*n* = 4), and the schematic diagram to illustrate the influence of the nanoporous and non-porous carrier on the DPI aerosolization performance (**c**).

Concentration of budesonide aerosolized from different DPI formulations was depicted in Figure 4. The resultant pharmacokinetic parameters were displayed in Tables 2 and 3. The trends in the pharmacokinetic profile of lung (Figure 4a) mirror those of plasma (Figure 4b). The budesonide concentration reached to a maximum value after 10 min (T_max_ = 10 min), then declined until minor or no budesonide was detected in the plasma and lung up to 8 h. The amount of budesonide deposited in the lung and absorbed into the blood for all formulations followed the rank order of M_4_ > Pulmicort > M_0_. The budesonide concentration of lung was far more than that of plasma, suggesting a promising local treatment for asthma and reduced risk of undesired systemic effects. This *in vivo* deposition result also agreed with the *in vitro* deposition study. Therefore, a good *in vitro-in vivo* correlation of drug lung delivery efficiency was established despite of the complexity of *in situ* respiratory system.

*C*_max_ of lung budesonide concentration for M_0_, M_4_ and Pulmicort was 75.29 ± 10.83 μg/g, 149.94 ± 15.27 μg/g, 95.95 ± 6.91 μg/g, respectively. The highest AUC_0-8h_ was found in M_4_, which showed a 2-fold AUC_0-8h_ (297.89 μg·h/mL) of Pulmicort (153.03 μg·h/mL), and a 3.2-fold of M_0_ (92.76 μg·h/mL). It was demonstrated that the highest drug delivery efficiency was achieved by using nanoporous particles as carriers. The significant improvement of *in vivo* aerosolization performance was achieved with nanoporous mannitol, which might be ascribed to the major advantages of pores in nanoscale conferred to the carrier. As shown in Figure 4c, the non-porous particles with smooth surface had relatively small contact area with the drug, and consequently generated low interparticulate force with the drug. In this case, the drug had a high tendency to detach from the carrier particle and deposite upon the mouth and pharynx before reaching the bronchus, resulting in reduced lung deposition. Unlike the non-porous particles, the nanoporous particles with small asperities could provide proper contact area for the drug binding, which ensured easy drug-carrier detachment upon the bronchus rather than the mouth and pharynx. Therefore, the nanoporous particles have greater potential to deliver the drug into the deep lung, and thus attain better aerosolization performance. Pulmicort is a commercial product, and only consists micronized budesonide^22^. In clinics, the delivery of Pulmicort is performed by using Turbohaler as the inhalation device. Turbohaler is a high resistance device operated under turbulent airflow to disperse powder, and reported to produce a superior FPF value by decreasing oropharyngeal deposition^22^. It is thus postulated that the M_4_ DPI formulation, if formulated in the Turbohaler, could potentially achieve a satisfying drug lung deposition efficiency. Additionally, the short time for the M_4_ DPI formulation to reach peak (*T*_max_ = 10 min) and half-life (*T*_1/2_ = 156.66 min) was indicative of a rapid onset and easy elimination of budesonide through inhalation. Such a dosage form meets the requirement of patients with acute asthma.

## Conclusions

Nanoporous mannitol was successfully prepared from a non-organic solvent system by one-step spray drying. The TGA and XPS analysis indicated that no ammonium carbonate existed in the mannitol. Therefore, this method demonstrated to be a green approach due to free of organic solvents and additives. The particle density rather than other physicochemical properties of mannitol was easily altered by adjusting the concentration of ammonium carbonate. A good linear correlation between the carrier density and FPF (*r*^*2*^ = 0.9579) was established, which confirmed the feasibility of using porous mannitol as model carrier density to investigate the influence of carrier density on the aerosolization performance of DPI. Compared to the non-porous mannitol, the nanoporous mannitol showed significantly decreased particle density, increased SSA, and rougher surface. This unique nature of nanoporous mannitol made it capable to remarkably enhance the deposition efficiency *in vitro* and *in vivo* by reducing the drug-carrier interaction sites and facilitating the drug-carrier detachment. In conclusion, carrier surface roughness modification by preparing nanoporous particle from the non-organic solvent system was proved to be an effective approach to enhance the aerosolization performance of DPI.

## Methods

### Preparation of porous and non-porous mannitol

**Figure 5:**
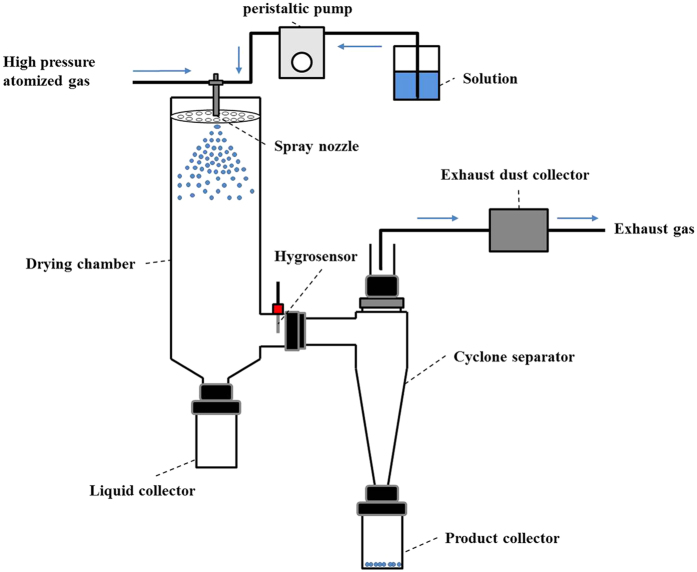
The schematic diagram of spray drier.

All solutions were spray dried by a laboratory-scale spray-dryer (Eyela SD-1000, Tokyo Rikakikai Co., Ltd., Japan) (Figure 5). The solutions were obtained by dissolving mannitol with or without ammonium carbonate in deionized water, and maintained the solid concentration of 2.5% (w/v). In all cases, the manufacture parameters were: nozzle tip 0.71 mm, feed rate 10 mg/min, and gas flow rate 0.60 m^3^/min.

The outlet temperature of 65 °C, 75 °C, and 85 °C was used to study their effect on the morphology of mannitols when the ratio of mannitol and ammonium carbonate was set as 3:1 (w/w). To study the concentration of ammonium carbonate on the characteristics of mannitols, the outlet temperature was maintained at 75 °C, and the ratio of mannitol and ammonium carbonate was set as 7:1, 5:1, 4:1, 3:1, 2:1 and 1:1 (w/w), respectively. The spray-dried mannitols were correspondingly labeled as M_1_, M_2_, M_3_, M_4_, M_5_, and M_6_. Mannitol alone, without adding ammonium carbonate, was spray dried under the same condition to act as a comparision (M_0_).

### Characterizations of spray-dried mannitols

#### Morphology

Scanning electron micrographs of the spray-dried mannitols were taken using a field emission scanning electron microscope (JSM-6330F, Japan) under a voltage of 15.0 kV. Samples were fixed on aluminium stubs and sputter-layered with gold prior to visualization.

#### Particle size analysis

The particle size of spray-dried mannitols was determined by laser diffraction (Malvern 2000, Malvern Instruments, Malvern, UK). The particles were dispersed using the Scirocco 2000 dry powder feeder at a dispersive air pressure of 3.5 bars. All samples were performed in triplicate with the obscuration values between 0.5% and 5%.

#### Bulk and tap density

The bulk density (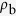) and tap density (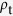) of raw and spray-dried mannitol were measured as previously described^31^. Samples were filled into a 1 ml graduated syringe, and the volume was recorded as bulk volume. The syringe was tapped onto a hard bench until no volume was changed, and then tapped volume was recorded. Each measurement was performed in triplicate. Bulk and tap density were calculated as the ratio of the mass to the bulk volume and tapped volume, respectively.

#### Specific surface area and pore size

A surface area and pore size analyzer (QuadraSorb eva, U.S.A.) was used to determine the specific surface area (SSA) of spray-dried mannitols. All samples were degassed under vacuum at 40 °C for 12 hours prior to measurement. The SSA was calculated according to the multiple-point Brunauer–Emmett– Teller (BET) equation. The pore size of optimal mannitol with regular pores was further determined by this apparatus.

#### Ammonium carbonate residues assay

The thermogravimetric analysis (TGA) and X-ray photoelectron spectroscopy (XPS) detection were conducted to determine whether ammonium carbonate was completely removed from the spray-dried mannitols.

TGA analysis (STA 409PC, Netzsch, Germany) was performed to record the mass loss between 40 °C to 80 °C to determine ammonium carbonate residues since ammonium carbonate could degrade above 60 °C.

XPS is a technique to detect the presence of chemical elements located less than 5 nm below the particle surface^32^. Therefore, the N1s photoelectron, which only exists in the ammonium carbonate was used as an indication of ammonium carbonate residues. The XPS measurements were performed on an XPS apparatus (VG Escalab 250 iXL ESCA, VG Scientific, U. K.) to obtain high-resolution spectra for the C 1 s, O 1 s, and N1s photoelectrons. X-rays were provided by an aluminum Kα (1486.6 eV) y source. All measurements were operated at a power of 100 W with an anode voltage of 15 kV and a current of 6.7 mA.

### *In vitro* and *in vivo* deposition studies

#### *In vitro* aerosolization performance

Micronized budesonide (98.5%, Yi Chang, China) with a *d*_0.5_ value of 2.303 ± 0.24 μm obtained by jet mill (YoutePowder Mechanical Equipment Co., Ltd, Yixing, China) was manually mixed with spray-dried mannitols (M_0_–M_6_) at a ratio of 1:30 (w/w), and filled into Size 3 hard hydroxypropyl methylcellulose (HPMC) capsule (Suzhou Capsugel Co., Ltd., China). Each capsule contained 10.0 ± 0.5 mg powder, corresponding to 33.3 ± 0.5 μg of budesonide. The capsules were sealed in aluminum foil before analysis.

The aerosolization performance of different DPI formulations and commercial product Pulmicort was determined using apparatus E (next generation impactor, NGI, Copley Instruments Ltd., UK) and operated under standard conditions (European Pharmacopoeia 7.0). Each capsule was loaded into a single dose inhaler Turbospin (PH&T, Milan, Italy), followed by impaction study through the NGI at a flow rate of 60 L/min for 4 s and a pressure drop of 4 KPa across the device. This operation was repeated five times. The collection stages of the impactors were washed with ethanol to determine budesonide concentration. Budesonide in the recovered solution was quantified spectrophotometrically at 240 nm by an HPLC system (LC-20, Shimadzu Co., Ltd.) equipped with an ODS column (Luna, 5 μm, 250 × 4.6 mm, Phenomenex). The mobile phase (acetonitrile : water = 70:30, v/v) was run at a speed of 1.0 ml/min.

The FPF was defined as the total percent of drug deposited in stage 1 and lower (≤4.46 μm) relative to the loaded dose. Each formulation was performed in triplicate.

### *In vivo* pharmacokinetic studies

#### Animals

Sprague-Dawley rats (180–220 g, male) were procured from the Experiment Animal Center of Guangzhou University of Chinese Medicine. All animal experiments were carried out with the permission of the Ethical Committee of Sun Yat-Sen University (Applicant number, IACUC-DD-16-0706) and performed in accordance with the Code of Ethics of the World Medical Association. The rats were fasted overnight prior to the experiments.

#### Dosing protocol

The rats (n = 120) were averagely divided into three groups, and anesthetized using 20% v/v urethane solution (5 ml/kg) by intraperitoneal injection before endotracheal administration of testing formulations. Group 1, Group 2, and Group 3 were administered the DPI formulations DPI_1_ (M_0_ used as the carrier), DPI_2_ (M_4_ used as the carrier), and Pulmicort, respectively. The first trachea bifurcation (carina) of rats was exposed by surgery before endotracheal administration. DP-4R dry powder insufflator (Pen-century Inc. Wyndmoor, PA, USA) was used to generate aerosol particles. A precisely weighted quantity of DPI, equivalent to 870 μg/kg body weight of budesonide, was loaded into the DP-4R sample chamber. The delivery tube of insufflator was introduced to the trachea just before carina as previously described^33^. The dry powder insufflation was performed by connecting the insufflator to a 3-ml air syringe with a plunger from a 1.5-ml air syringe, which corresponded to the tidal volume of rats. Approximately 90% of the metered dose could be delivered into the tract of rats after five repeated insufflation. After administration, four rats were sacrificed at 0 min, 5 min, 10 min, 15 min, 30 min, 45 min, 1 h, 2 h, 4 h, and 8 h, respectively. Blood samples were collected by cardiac puncture in heparinized tubes, and lungs were isolated to obtain lung homogenate at predetermined time intervals. The plasma and isolated lung were stored at −80 °C until analysis.

#### Lung homogenate and plasma preparation

The lungs were homogenized by a homogenizer (IKA T10, Germany) in an ice bath to obtain lung homogenate. Budesonide was extracted by liquid-liquid extraction of the lung homogenate and plasma. Initially, a 500 μl of homogenate or plasma was added to a 10-ml tube containing 200 μl internal standard (triamcinolone acetonide), and vortex mixed for 2 min. Subsequently, 10 μl 0.1 mg/ml of acetic acid solution and 2 ml ethyl acetate were added and vortex mixed for 10 min. The suspension was centrifuged using a TGL-16C desktop centrifuge (Shanghai Anting Scientific Instruments Factory, Shanghai, China) for 10 min at 3000 r/min to obtain the supernatant. The extraction procedure was repeated twice. The combined supernatant was vacuum dried and re-dissolved with 200 μl mobile phase (methanol: 0.5% v/v formic acid solution = 70:30).

#### HPLC-MS analysis

Budesonide concentration in the plasma and lung was determined by HPLC-MS. The mass spectrometer source (TSQ Quantum, Finnigan) was operated in positive mode. The detection ions were 413.37-147.40 (budesonide) and 397.30-213.30 (triamcinolone acetonide).

### Statistical analysis

Statistical analysis was performed to the data obtained from pharmacokinetic study using IBM SPSS Statistics 22.0 software (IBM Corporation, Armonk, NY, USA). Data were reported as mean ± SD. All of the data were firstly examined their skewness by Shapiro-Wilk’s tests and homogeneity of variance by Levene’s tests. Unambiguously, only when insignificant results were confirmed in these two tests simultaneously, one-way ANOVA followed by LSD’s post hoc analysis was launched to compare data from different groups. Otherwise, Kruskal-Wallis’ tests followed by Bonferroni’s post hoc analysis were carried out for pairwise multiple comparisons. Significance level was set at P < 0.05 in all the tests.

## Additional Information

**How to cite this article:** Peng, T. *et al*. Nanoporous mannitol carrier prepared by non-organic solvent spray drying technique to enhance the aerosolization performance for dry powder inhalation. *Sci. Rep.* **7**, 46517; doi: 10.1038/srep46517 (2017).

**Publisher's note:** Springer Nature remains neutral with regard to jurisdictional claims in published maps and institutional affiliations.

## Supplementary Material

Supplementary Information

## Figures and Tables

**Table 1:** The particle size, bulk density (*ρ*_b_), tap density (*ρ*_t_), and specific surface area (SSA) of spray-dried mannitols prepared at different ratio of ammonium carbonate and mannitol (*n* = 3).

Mannitols	*d*_0.5_ (μm)	*ρ*_b_(g/cm^3^)	*ρ*_t_(g/cm^3^)	SSA (m^2^/g)	Pore diameter (nm)
M_0_	5.32 ± 0.08	0.39 ± 0.02	0.61 ± 0.05	1.62 (1.80, 1.43)	
M_1_	5.25 ± 0.21	0.16 ± 0.01	0.63 ± 0.03	2.63 (2.40, 2.87)	
M_2_	5.48 ± 0.96	0.13 ± 0.00	0.63 ± 0.06	11.56 (10.23, 12.89)	
M_3_	5.13 ± 0.43	0.12 ± 0.01	0.55 ± 0.04	25.35 (28.34, 22.35)	
M_4_	5.05 ± 0.05	0.09 ± 0.00	0.38 ± 0.03	42.29 (42.37, 42.21)	5.90
M_5_	4.93 ± 0.11	0.13 ± 0.01	0.57 ± 0.05	17.61 (19.99, 15.23)	
M_6_	5.09 ± 0.32	0.18 ± 0.01	0.78 ± 0.00	6.11 (5.23, 6.99)	

**Table 2:** The pharmacokinetic parameters of different DPI formulations in the lung (*n* = 4). Data were reported as mean ± SD.

Formulations	C_max_ (ug/g)	T_max_ (min)	AUC_0-8h_ (ug·h/g)	AUC_0~∞_D_ (kg·h/g)	MRT_0-8h_ (min)	T_1/2_ (min)
M_0_	75.29 ± 10.83	10	92.67 ± 6.90	0.10 ± 0.01	108.66 ± 2.01	109.67 ± 2.61
M_4_	149.94 ± 15.27^*,#^	10	297.89 ± 16.02^*,#^	0.40 ± 0.03^*,#^	158.85 ± 4.34^*^	156.66 ± 8.68^*^
Pulmicort	95.95 ± 6.91	10	153.03 ± 10.71^*^	0.10 ± 0.01	153.03 ± 10.71^*^	177.69 ± 21.95^*^

Comparison between groups was done by one way AVOVA followed by Bonferroni post hoc test.

^*^p  < 0.05 *vs.* M_0_ and ^#^*p* < 0.05 *vs*. Pulmicort.

**Table 3:** The pharmacokinetic parameters of different DPI formulations in the plasma (*n* = 4). Data were reported as mean ± SD.

Formulations	C_max_ (ug/ml)	T_max_ (min)	AUC_0-8h_ (ug·h/ml)	AUC_0~∞_D_ (g·h/ml)	MRT_0-8h_ (min)	T_1/2_ (min)
M_0_	1.37 ± 0.06	10	1.11 ± 0.11	2.64 ± 0.49	96.89 ± 11.70	90.68 ± 5.11
M_4_	1.74 ± 0.12*	10	1.57 ± 0.13*^,#^	1.84 ± 0.15	79.05 ± 3.78*	156.04 ± 16.30*
Pulmicort	1.59 ± 0.14	10	1.30 ± 0.07	0.51 ± 0.04	79.46 ± 7.80*	169.87 ± 10.99*

Comparison between groups was done by one way AVOVA followed by Bonferroni post hoc test.

^*^p  < 0.05 *vs.* M_0_ and ^#^*p* < 0.05 *vs*. Pulmicort.

## References

[b1] Patil-Gadhe A. & Pokharkar V. Single step spray drying method to develop proliposomes for inhalation: A systematic study based on quality by design approach. Pulm Pharmacol Ther 27, 197–207, doi: 10.1016/j.pupt.2013.07.006 (2014). 23916767

[b2] Du P., Du J. & Smyth H. D. C. Evaluation of Granulated Lactose as a Carrier for DPI Formulations 1: Effect of Granule Size. Aaps Pharmscitech 15, 1417–1428, doi: 10.1208/s12249-014-0166-z (2014). 24962007 PMC4245420

[b3] Kaialy W. & Nokhodchi A. Treating mannitol in a saturated solution of mannitol: A novel approach to modify mannitol crystals for improved drug delivery to the lungs. Int J Pharm 448, 58–70, doi: 10.1016/j.ijpharm.2013.03.005 (2013). 23500603

[b4] Young P. M. . Composite carriers improve the aerosolisation efficiency of drugs for respiratory delivery. J Aerosol Sci 39, 82–93, doi: 10.1016/j.jaerosci.2007.10.003 (2008).

[b5] Adi S. . Effects of mechanical impaction on aerosol performance of particles with different surface roughness. Powder Technol 236, 164–170, doi: 10.1016/j.powtec.2012.02.051 (2013).

[b6] Zeng X. M., Martin G. P., Marriott C. & Pritchard J. The Influence of Crystallization Conditions on the Morphology of Lactose Intended for Use as a Carrier for Dry Powder Aerosols. J Pharm Pharmacol 52, 633–643, doi: 10.1211/0022357001774462 (2000). 10875539

[b7] Grasmeijer F. . New Mechanisms to Explain the Effects of Added Lactose Fines on the Dispersion Performance of Adhesive Mixtures for Inhalation. Plos One 9, e87825, doi: 10.1371/journal.pone.0087825 (2014). 24489969 PMC3905031

[b8] Guchardi R., Frei M., John E. & Kaerger J. S. Influence of fine lactose and magnesium stearate on low dose dry powder inhaler formulations. Int J Pharm 348, 10–17, doi: 10.1016/j.ijpharm.2007.06.041 (2008). 17689898

[b9] Traini D., Scalia S., Adi H., Marangoni E. & Young P. M. Polymer coating of carrier excipients modify aerosol performance of adhered drugs used in dry powder inhalation therapy. Int J Pharm 438, 150–159, doi: 10.1016/j.ijpharm.2012.08.036 (2012). 22964399

[b10] Littringer E. M. . The morphology and various densities of spray dried mannitol. Powder Technol 246, 193–200, doi: 10.1016/j.powtec.2013.05.004 (2013).

[b11] Notario B., Pinto J. & Rodriguez-Perez M. A. Nanoporous polymeric materials: A new class of materials with enhanced properties. Prog Mater Sci 78–79, 93–139, doi: 10.1016/j.pmatsci.2016.02.002 (2016).

[b12] Chiang Y.-D. . Controlling Particle Size and Structural Properties of Mesoporous Silica Nanoparticles Using the Taguchi Method. Journal Phys Chem C 115, 13158–13165, doi: doi: 10.1021/jp201017e (2011).

[b13] Wu K. C. W. & Yamauchi Y. Controlling physical features of mesoporous silica nanoparticles (MSNs) for emerging applications. J Mater Chem 22, 1251–1256, doi: 10.1039/C1JM13811A (2012).

[b14] Huang H.-S. . Evaporation-Induced Coating of Hydrous Ruthenium Oxide on Mesoporous Silica Nanoparticles to Develop High-Performance Supercapacitors. Small 9, 2520–2526, doi: 10.1002/smll.201202786 (2013). 23494855

[b15] Malgras V. . Templated Synthesis for Nanoarchitectured Porous Materials. Bull Chem Soc Jpn 88, 1171–1200, doi: 10.1246/bcsj.20150143 (2015).

[b16] Li B., Chrzanowski M., Zhang Y. & Ma S. Applications of metal-organic frameworks featuring multi-functional sites. Coordin Chem Rev 307, Part 2, 106–129, doi: 10.1016/j.ccr.2015.05.005 (2016).

[b17] Yamada T., Sadakiyo M., Shigematsu A. & Kitagawa H. Proton-Conductive Metal-Organic Frameworks. Bull Chem Soc Jpn 89, 1–10, doi: 10.1246/bcsj.20150308 (2016).

[b18] Iskandar F. . Production of morphology-controllable porous hyaluronic acid particles using a spray-drying method. Acta Biomater 5, 1027–1034, doi: 10.1016/j.actbio.2008.11.016 (2009). 19114316

[b19] Ógáin O. N., Li J., Tajber L., Corrigan O. I. & Healy A. M. Particle engineering of materials for oral inhalation by dry powder inhalers. I—Particles of sugar excipients (trehalose and raffinose) for protein delivery. Int J Pharm 405, 23–35, doi: 10.1016/j.ijpharm.2010.11.039 (2011). 21129458

[b20] Littringer E. M. . Spray Drying of Mannitol as a Drug Carrier—The Impact of Process Parameters on Product Properties. Dry Technol 30, 114–124, doi: 10.1080/07373937.2011.620726 (2012).

[b21] Littringer E. M. . The morphology of spray dried mannitol particles — The vital importance of droplet size. Powder Technol 239, 162–174, doi: 10.1016/j.powtec.2013.01.065 (2013).

[b22] Nolan L. M. . Excipient-free nanoporous microparticles of budesonide for pulmonary delivery. Eur J Pharm Sci 37, 593–602, doi: 10.1016/j.ejps.2009.05.007 (2009). 19463948

[b23] Kaialy W., Alhalaweh A., Velaga S. P. & Nokhodchi A. Influence of lactose carrier particle size on the aerosol performance of budesonide from a dry powder inhaler. Powder Technol 227, 74–85, doi: 10.1016/j.powtec.2012.03.006 (2012).

[b24] Tewes F. . Steroid/mucokinetic hybrid nanoporous microparticles for pulmonary drug delivery. Eur J Pharm Biopharm 85, 604–613, doi: 10.1016/j.ejpb.2013.03.020 (2013). 23563102

[b25] Ebrahimi A., Saffari M., Dehghani F. & Langrish T. Incorporation of acetaminophen as an active pharmaceutical ingredient into porous lactose. Int J Pharm 499, 217–227, doi: 10.1016/j.ijpharm.2016.01.007 (2016). 26768724

[b26] Zellnitz S., Redlinger-Pohn J. D., Kappl M., Schroettner H. & Urbanetz N. A. Preparation and characterization of physically modified glass beads used as model carriers in dry powder inhalers. Int J Pharm 447, 132–138, doi: 10.1016/j.ijpharm.2013.02.044 (2013). 23470233

[b27] Zhou Q. & Morton D. A. V. Drug–lactose binding aspects in adhesive mixtures: Controlling performance in dry powder inhaler formulations by altering lactose carrier surfaces. Adv Drug Delivery Rev 64, 275–284, doi: 10.1016/j.addr.2011.07.002 (2012). 21782866

[b28] Kaialy W. . The influence of physical properties and morphology of crystallised lactose on delivery of salbutamol sulphate from dry powder inhalers. Colloid Surface B 89, 29–39, doi: 10.1016/j.colsurfb.2011.08.019 (2012). 21962946

[b29] Ooi J., Traini D., Hoe S., Wong W. & Young P. M. Does carrier size matter? A fundamental study of drug aerosolisation from carrier based dry powder inhalation systems. Int J Pharm 413, 1–9, doi: 10.1016/j.ijpharm.2011.04.002 (2011). 21501674

[b30] Kaialy W., Martin G. P., Ticehurst M. D., Momin M. N. & Nokhodchi A. The enhanced aerosol performance of salbutamol from dry powders containing engineered mannitol as excipient. Int J Pharm 392, 178–188, doi: 10.1016/j.ijpharm.2010.03.057 (2010). 20363301

[b31] Healy A. M., McDonald B. F., Tajber L. & Corrigan O. I. Characterisation of excipient-free nanoporous microparticles (NPMPs) of bendroflumethiazide. Eur J Pharm Biopharm 69, 1182–1186, doi: 10.1016/j.ejpb.2008.04.020 (2008). 18595674

[b32] Hadinoto K., Phanapavudhikul P., Kewu Z. & Tan R. B. H. Dry powder aerosol delivery of large hollow nanoparticulate aggregates as prospective carriers of nanoparticulate drugs: Effects of phospholipids. Int J Pharm 333, 187–198, doi: 10.1016/j.ijpharm.2006.10.009 (2007). 17084567

[b33] Bivas-Benita M., Zwier R., Junginger H. E. & Borchard G. Non-invasive pulmonary aerosol delivery in mice by the endotracheal route. Eur J Pharm Biopharm 61, 214–218, doi: 10.1016/j.ejpb.2005.04.009 (2005). 16039104

