# Supplementary material for: Nanoporous mannitol carrier prepared by non-organic solvent spray drying technique to enhance the aerosolization performance for dry powder inhalation
Source: Sci Rep. 2017 May 2;7:46517. doi: 10.1038/srep46517 (PMC5411962; doi:10.1038/srep46517)
Supplement: Supplementary Information [file srep46517-s1.doc]

**Title page**

**Nanoporous mannitol carrier prepared by non-organic solvent spray drying technique to enhance the** **aerosolization performance for dry powder inhalation**

Tingting Peng^1, #^, Xuejuan Zhang^1, #^, Ying Huang^1^, Ziyu Zhao^1^, Qiuying Liao^1^, Jing Xu^1^, Zhengwei Huang^1^, Jiwen Zhang^2^, Chuan-yu Wu^3^, Xin Pan^1, 4^, *, Chuanbin Wu^1, 5^, *

^1^ School of Pharmaceutical Sciences, Sun Yat-Sen University, Guangzhou 510006, China;

^2^ Shanghai Institute of Materia Medica, Chinese Academy of Sciences,

Shanghai 201203, China;

^3^ Department of Chemical and Process Engineering, University of Surrey, Guildford,

Surrey GU2 7XH, United Kingdom;

^4^ Zhongshan WanYuan New Drug R&D Co., Ltd., Zhongshan City 528451, China;

^5^ Guangdong Research Center for Drug Delivery Systems, Guangzhou 510006, China

^#^ The first two authors contributed equally to this work.

^＊^Corresponding author:

Xin Pan, Ph.D. Tel: +862039943427; Fax: +862039943115; E-mail: [pxin_1385@163.com](mailto:pxin_1385@163.com)

School of Pharmaceutical Sciences, Sun Yat-Sen University, Guangzhou 510006, China

Chuanbin Wu, Professor. Tel: +862039943120; Fax: +862039943115;

E-mail: [chuanbin_wu@126.com](mailto:chuanbin_wu@126.com)

School of Pharmaceutical Sciences, Sun Yat-Sen University, Guangzhou 510006, China

**Solid state characterization of spray-dried mannitols**

The crystalline form of spray-dried and raw mannitols was determined by DSC, PXRD, and FT-IR.

As shown in Fig. 6a, the DSC traces of all mannitols displayed comparable distinctive endothermic transitions at 165~166°C, corresponding to the melting of α and/or β mannitol. Indeed, α-mannitol and β –mannitol forms are distinguishable in DSC traces since their melting points are nearly similar. Therefore, the solid state form of all mannitols was further analyzed by PXRD and FT-IR.

The PXRD patterns (Fig. 6b) showed that all mannitols exhibited the diagnostic peaks (10.6º, 14.7 º, 23.4 º, and 29.5 º) of β–mannitol. This was supportive to FT-IR results since the mannitols exhibited the IR specific bands (929 cm^−1^, 959 cm^−1^, and 1,029 cm^−1^) of diagnosis peak β–mannitol (Fig. 6c).

Overall, the crystalline evaluation showed that all mannitiols were in the β crystalline form regardless of adding ammonium carbonate during spray drying.


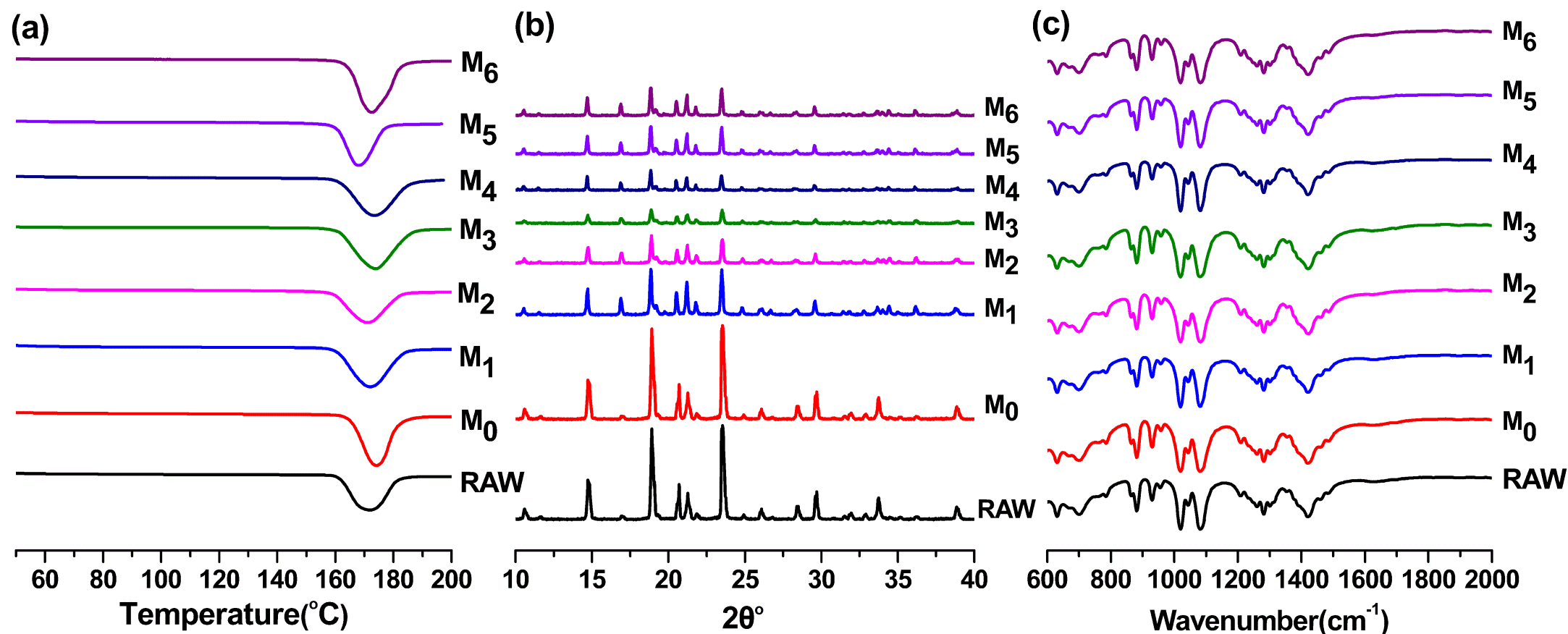


Fig. 6: Solid state of spray-dried and raw mannitols characterized by different methods: (a) DSC curves, (b) XRD diffractograms, and (c) FT-IR spectra.
